# The efficacy and safety of telitacicept and its combination with glucocorticoids in IgA nephropathy: a retrospective cohort study

**DOI:** 10.3389/fphar.2026.1821538

**Published:** 2026-05-29

**Authors:** Yiyang Li, Lijuan Zhao, Meijing Pan, Yan Wang, Xin Gou, Xinrong Yang, Qian Zhang, Chenchen Ma, Jiaoyang Zhang, Guoshuang Xu

**Affiliations:** 1 Department of Nephrology, Xijing Hospital, Fourth Military Medical University, Xi’an, China; 2 The Graduate School of Xi’an Medical University, Xi’an, China

**Keywords:** efficacy, IgA nephropathy, proteinuria, safety, telitacicept

## Abstract

**Background:**

Immunoglobulin A nephropathy (IgAN) has a high incidence rate and a poor prognosis. Currently, no ideal treatment is available for this disease. Despite some reports on the use of telitacicept in IgAN, studies with a treatment duration of 24 weeks or longer remain limited, and data on its combination with glucocorticoids are currently lacking.

**Methods:**

This retrospective cohort study enrolled 136 individuals diagnosed with IgAN via renal biopsy who exhibited 24-h urinary protein excretion (UPE) ≥ 1 g and an estimated glomerular filtration rate (eGFR) ≥ 30 ml/min/1.73 m^2^. Of these, 25 received RAS inhibitors, 55 received glucocorticoid ± immunosuppressants, 18 received telitacicept, and 38 received telitacicept + glucocorticoids.

**Results:**

At 24 weeks, compared with baseline, median UPE decreased by 0.70 g (41.75%) in the RAS inhibitor group, by 0.92 g (62.85%) in the glucocorticoid ± immunosuppressant group, by 1.14 g (73.68%) in the telitacicept group, and by 1.34 g (81.18%) in the telitacicept + glucocorticoid group. The reduction in UPE was significantly greater in the latter three groups than in the RAS inhibitor group (p < 0.05). Moreover, the telitacicept + glucocorticoid group demonstrated a significantly greater reduction in UPE than the other groups. The eGFR decreased by 3.22 ml/min/1.73 m^2^ (−16.77, −0.70) in the RAS inhibitor group compared with baseline, whereas it remained stable in the other groups. Among patients with UPE ≥2.5 g or eGFR <60 ml/min/1.73 m^2^, the telitacicept + glucocorticoid group showed a significantly greater reduction in UPE than the other groups (p < 0.05).

**Conclusion:**

These findings indicate that in IgAN patients with a high risk of progression, telitacicept effectively reduces proteinuria and confers kidney protective effects. Furthermore, in individuals with UPE ≥2.5 g or impaired renal function, the combination of telitacicept and glucocorticoids is associated with improved rates of UPE remission.

## 1 Introduction

Immunoglobulin A Nephropathy (IgAN) is the most prevalent primary glomerular disease worldwide. Its pathological hallmark is the deposition of IgA, predominantly in the glomerular mesangial region, often accompanied by other immunoglobulins, leading to renal tissue damage (Floege and Amann, 2016; Pattrapornpisut et al., 2021; Stamellou et al., 2023). The global incidence of IgAN exceeds 2.5 cases per 100,000 individuals, with particularly high rates in Asian countries. The 10-year kidney survival rate is only 60%–80% (Schena and Nistor, 2018; Cheung et al., 2025; Shen et al., 2025). It is also a significant cause of chronic kidney disease (CKD) and kidney failure among young people, with substantial regional variations (Wong et al., 2024). China is among the countries with the highest global incidence rates of IgAN. The disease typically manifests at a relatively young age, with a median age of onset of 34 years. The prognosis remains poor, as approximately 60% of patients progress to end-stage kidney disease (ESKD) within 15 years (Stamellou et al., 2023; Shen et al., 2025).

The pathogenesis of IgAN remains unclear, with the most widely accepted explanation being the “4-hit” hypothesis (Suzuki and Novak, 2021; Zachova et al., 2022). Galactose-deficient IgA1 (Gd-IgA1) is a key pathogenic factor of IgAN. The serum levels are closely associated with disease occurrence and progression (Shi et al., 2025). Mucosa-associated lymphoid tissue (MALT) activation drives production of Gd-IgA1 and anti-Gd-IgA1 autoantibodies. This triggers an immune response and stimulates inflammation, promoting B cell proliferation and activation (Gesualdo et al., 2021). B lymphocyte stimulator/B-cell activating factor (BLyS/BAFF) and proliferation-inducing ligand (APRIL) are key regulators of B-cell activation. They bind to the transmembrane activator and calmodulin cyclin ligand interaction factor (TACI), B cell maturation antigen (BCMA), and BAFF receptor to promote B cell maturation, differentiation, and immunoglobulin production. This process drives the generation of Gd-IgA1 and leads to the development of IgAN (Xin et al., 2013; Cheung et al., 2023; Mathur et al., 2023).

In recent years, therapeutic strategies targeting BAFF and APRIL have demonstrated preliminary efficacy in the treatment of IgAN (Floege et al., 2025). Among these approaches, telitacicept represents a promising agent that modulates B-cell activation through dual suppression of BAFF and APRIL signaling pathways (Samy et al., 2017). Telitacicept is a fully human recombinant fusion protein composed of the extracellular domain of TACI fused to the Fc portion of human immunoglobulin G (Dhillon, 2021). It functions as a soluble decoy receptor that competitively binds to both BAFF and APRIL, thereby blocking their interaction with receptors on B cells, including BCMA, TACI, and BAFF receptor (Chang and Li, 2020). This dual blockade suppresses the survival, maturation, and differentiation of autoreactive B cells and plasma cells, which are key sources of Gd-IgA1 in IgAN. By reducing circulating levels of Gd-IgA1 and the formation of immune complexes, telitacicept mitigates their deposition in the glomerular mesangium and subsequent local inflammatory injury, offering a targeted therapeutic strategy for IgAN (Samy et al., 2017). A Phase II clinical study of telitacicept in patients with IgAN and persistent proteinuria demonstrated a higher rate of urinary protein remission compared with standard therapy, particularly with renin-angiotensin system (RAS) inhibitors (Shi et al., 2021; Lv et al., 2023). Moreover, telitacicept can significantly reduce circulating Gd-IgA1 levels (Zan et al., 2024).

Managing glomerular inflammation (such as systemic low-dose glucocorticoids) and traditional immunosuppressants (such as mycophenolate mofetil) also demonstrate considerable efficacy in IgAN (Lv et al., 2022; Hou et al., 2023; Chen and Lv, 2024). However, in this high-risk type of IgAN, there remains a lack of robust evidence comparing telitacicept with traditional immunosuppressants therapy. In this study, we compare the efficacy and safety of telitacicept monotherapy, telitacicept combined with a low-dose glucocorticoid, RAS inhibitor, and glucocorticoid ± immunosuppressant therapy in the treatment of IgAN.

## 2 Materials and methods

### 2.1 Study population

This was a single-center, retrospective cohort study. The research subjects were individuals with IgAN who were followed up at Xijing Hospital from October 2023 to December 2024.

Inclusion criteria are as follows: (1) Primary IgAN confirmed by renal biopsy; (2) Age between 18 and 70 years; (3) 24-h urinary protein excretion (UPE) ≥ 1 g; (4) Estimated glomerular filtration rate (eGFR) [calculated using the Chronic Kidney Disease Epidemiology and Prevention (CKD-EPI) formula] ≥ 30 ml/min/1.73 m^2^.

Exclusion criteria include: (1) Individuals with secondary IgAN, including but not limited to IgA vasculitis, systemic lupus erythematosus (SLE), infections, chronic liver disease, and malignant tumors, etc.,; (2) Individuals with incomplete baseline or follow-up data; (3) Individuals receiving other biological agents with immunosuppressive effects (including rituximab, etc., but excluding telitacicept); (4) Patients who had received treatment for less than 6 months; (5) Women who are pregnant or lactating.

### 2.2 Study design

We assigned patients who met the selection criteria to one of four treatment groups according to their therapeutic regimen: the RAS inhibitor group, which received angiotensin-converting enzyme inhibitors (ACEI) or angiotensin II receptor blockers (ARB) treatment; the glucocorticoid ± immunosuppressant group received prednisone or methylprednisolone ± cyclophosphamide or mycophenolate mofetil treatment; the telitacicept group received telitacicept only; the telitacicept + glucocorticoid group received the combined treatment of telitacicept and glucocorticoids simultaneously. During the treatment period, all patients received the maximum tolerated dose of the RAS inhibitors. The observation period was 24 weeks.

### 2.3 Drug administration

Glucocorticoid dosing was standardized as prednisone at a maximum of 0.5 mg/kg/day (with a ceiling dose of 40 mg) or methylprednisolone at a maximum of 0.4 mg/kg/day (with a ceiling dose of 32 mg). The dosage was gradually tapered after 4 weeks of initiation and discontinued by week 24. Cyclophosphamide was administered intravenously at 0.8 g once monthly, whereas mycophenolate mofetil was given orally at 1.0–1.5 g daily, divided into two doses. Telitacicept was administered subcutaneously at a dose of 240 mg/week.

### 2.4 Date collection

Demographic and clinical data were collected for each patient. Demographic information included age, sex, body mass index (BMI), medical history, Oxford histological score, and prior treatment regimens. Clinical data included baseline characteristics such as UPE, serum creatinine, serum total protein, serum albumin, aminotransferases, hemoglobin, total cholesterol, and triglycerides. We calculated the eGFR using the CKD-EPI formula. Collect UPE, urinary red blood cell (RBC) count, serum creatinine, and eGFR at 4, 8, 12, 16, 20, and 24 weeks. Adverse reactions were also recorded.

### 2.5 Endpoint

The primary endpoints of this study were the median and percentage changes in UPE from baseline during the treatment period. Secondary endpoints included changes in serum creatinine, eGFR, and urinary RBC count from baseline, along with a safety assessment.

## 3 Statistical analysis

Categorical data were expressed as numbers and percentages. For continuous variables, normally distributed data are presented as mean ± standard deviation (SD). Results for continuous data that do not follow a normal distribution are reported as the median, upper quartile, and lower quartile. Differences in characteristics among groups were compared using the independent-samples t-test when data were normally distributed and the Wilcoxon-Mann-Whitney test when data were not. The chi-square test was used to evaluate differences in categorical variables. All analyses were performed using SPSS Statistics version 27.0. Statistical significance was set at p < 0.05.

## 4 Results

### 4.1 Study population

**FIGURE 1:**
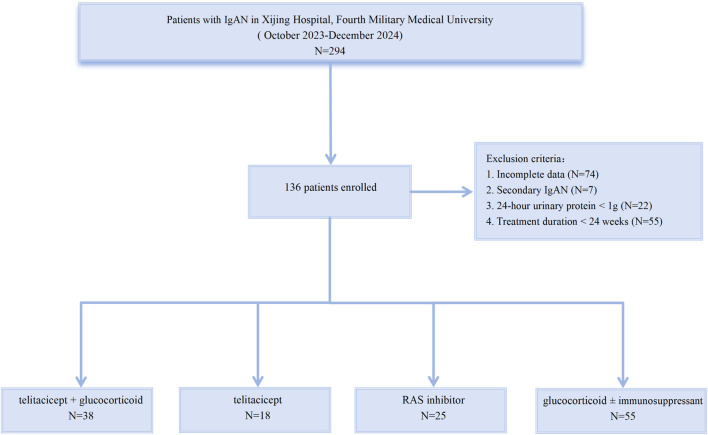
Flowchart of study design. IgAN, IgA nephropathy.

A total of 294 patients with IgAN were screened. We excluded 74 patients with incomplete data, 7 with secondary IgAN, 22 with UPE <1 g, and 55 with a treatment duration of <24 weeks. A total of 136 cases were ultimately included, among which 25 cases were in the RAS inhibitor group, 55 cases in the glucocorticoid ± immunosuppressant group, 18 cases in the telitacicept group, and 38 cases in the telitacicept + glucocorticoid group (Figure 1). In the glucocorticoid ± immunosuppressant group, 18 patients received glucocorticoid only, 14 patients received glucocorticoid combined with mycophenolate mofetil. The remaining 23 patients received glucocorticoid combined with cyclophosphamide.

### 4.2 Baseline characteristics

Table 1 summarizes the baseline characteristics of all individuals. The average age of all participants was 39 years, with males accounting for 52.2%. The middle UPE was 1.52 (1.18, 2.22) g, serum creatinine was 97.0 (66.0, 140.0) µmol/L, and eGFR was 75.62 (46.43, 109.39) ml/min/1.73 m^2^. Demographic and clinical characteristics, including age, sex, BMI, UPE, urinary RBC count, serum creatinine, eGFR, peripheral blood white blood cell count, hemoglobin, platelet count, transaminase level, fasting blood glucose, and uric acid, were comparable in all groups (all p > 0.05). Serum total protein level in telitacicept + glucocorticoid group, in telitacicept group, and in glucocorticoid ± immunosuppressant group was significantly lower than that in the RAS inhibitor group (65.96 ± 7.13, 67.16 ± 7.77, 66.12 ± 7.64 vs. 71.72 ± 5.80 g/L, p < 0.05, respectively). Concurrently, the serum albumin level in the RAS inhibitor group exhibited significantly higher than that in the telitacicept + glucocorticoid, telitacicept, and the glucocorticoid ± immunosuppressant groups (41.64 ± 4.49 vs. 37.60 ± 5.57, 38.32 ± 4.02, 37.69 ± 5.78 g/L, all p < 0.05, respectively). Compared with the glucocorticoid ± immunosuppressant group, baseline total cholesterol and triglyceride were lower in telitacicept + glucocorticoid group (4.32 ± 0.69 vs. 4.72 ± 1.01 mmol/L and 1.63 ± 0.54 vs. 2.02 ± 1.07 mmol/L, all p < 0.05). Oxford classification analysis revealed no statistically significant differences among the four groups in the distribution of active pathological lesions, including mesangial hypercellularity (M), endocapillary hypercellularity (E), segmental glomerulosclerosis (S), and cellular/fibrocellular crescents (C) (all P > 0.05). However, a significant intergroup difference was observed in the chronicity index of tubular atrophy/interstitial fibrosis (T) (P = 0.049). The RAS inhibitor group exhibited the highest proportion of T0 stage (76.0%) and the lowest proportion of T2 stage (8.0%). In contrast, the proportions of T2 stage were markedly higher in the glucocorticoid ± immunosuppressant group (20.0%), telitacicept group (22.2%), and telitacicept + glucocorticoid group (23.7%), respectively.

**TABLE 1:** Baseline characteristics of the participants with IgAN.

Variables	RAS inhibitor N = 25	glucocorticoids ± immunosuppressant N = 55	telitacicept N = 18	telitacicept + glucocorticoid N = 38	P
Age, yr	40.84 ± 11.99	38.16 ± 11.84	40.33 ± 13.76	39.34 ± 12.54	0.81
Female	12 (48.0%)	25 (45.5%)	9 (50.0%)	19 (50.0%)	0.29
BMI	24.37 ± 3.39	24.86 ± 3.91	25.90 ± 2.20	24.65 ± 2.98	0.51
Urinary protein, g/day	1.46 (1.17,2.37)	1.51 (1.11,2.00)	1.54 (1.22,2.50)	1.69 (1.18,2.24)	0.81
serum creatinine, μmol/L	79.00 (67.50,132.00)	97.00 (67.00,142.00)	88.50 (65.00,143.50)	124.00 (62.00,156.25)	0.35
eGFR, ml/min/1.73m^2^	91.60 (48.94,116.46)	76.37 (51.42,111.02)	91.03 (41.81,109.39)	56.01 (40.01,103.02)	0.40
Leukocyte,×109/L	6.37 (5.28,8.42)	6.62 (5.73,7.80)	6.64 (5.73,7.84)	7.38 (5.43,8.53)	0.32
Hemoglobin, g/L	141.00 (123.50,157.50)	151.00 (132.00,166.00)	152.50 (142.00,162.75)	153.50 (138.75,164.25)	0.26
Platelet, ×109/L	247.12 ± 73.85	239.40 ± 62.98	219.50 ± 59.30	226.26 ± 57.43	0.40
Aminotransferase, IU/L	19.32 ± 10.87	22.33 ± 11.99	26.61 ± 12.23	24.84 ± 11.13	0.15
Total cholesterol, mmol/L	4.46 ± 0.75	4.72 ± 1.01	4.42 ± 0.744	4.32 ± 0.69^#^	0.13
Triglyceride, mmol/L	1.79 ± 1.09	2.02 ± 1.07	1.69 ± 0.59	1.63 ± 0.54^#^	0.20
Serum total protein, g/L	71.72 ± 5.80	66.12 ± 7.64 *	67.16 ± 7.77 *	65.96 ± 7.13 *	0.01
Serum albumin, g/L	41.64 ± 4.49	37.69 ± 5.78 *	38.32 ± 4.02 *	37.60 ± 5.57 *	0.01
Urea, mmol/L	5.92 (4.53,8.25)	6.34 (5.28,8.29)	5.60 (4.08,8.18)	7.36 (5.08,9.30)	0.20
Uric acid, μmol/L	380.32 ± 100.14	404.75 ± 88.01	339.44 ± 82.80	365.54 ± 115.01	0.06
Urinary RBC count	71.90 (9.80,228.65)	119.80 (37.40,119.80)	167.95 (93.75,341.45)	59.00 (28.38,170.00)	0.88
Oxford classification, n (%)					
M	​	​	​	​	0.260
M0	13 (52.0%)	25 (45.5%)	7 (38.9%)	11 (28.9%)	​
M1	12 (48.0%)	30 (54.5%)	11 (61.1%)	27 (71.1%)	​
E	​	​	​	​	0.089
E0	13 (52.0%)	36 (65.5%)	13 (72.2%)	31 (81.6%)	​
E1	12 (48.0%)	19 (34.5%)	5 (27.8%)	7 (18.1%)	​
S	​	​	​	​	0.266
S0	8 (32.0%)	7 (12.7%)	3 (16.7%)	5 (13.2%)	​
S1	17 (68.0%)	48 (87.3%)	15 (83.3%)	33 (86.8%)	​
T	​	​	​	​	0.049
T0	19 (76.0%)	25 (45.5%)	7 (38.9%)	12 (31.6%)	​
T1	4 (16.0%)	19 (34.5%)	7 (38.9%)	17 (44.7%)	​
T2	2 (8.0%)	11 (20.0%)	4 (22.2%)	9 (23.7%)	​
C	​	​	​	​	0.097
C0	12 (48.0%)	17 (30.9%)	5 (27.8%)	22 (57.9%)	​
C1	12 (48.0%)	36 (65.5%)	12 (66.7%)	13 (34.2%)	​
C2	1 (4.0%)	2 (3.6%)	1 (5.6%)	3 (7.9%)	​
Previous medication, n (%)					
Glucocorticoid, n (%)	0	2 (3.64%)	11 (61.11%)	27 (68.42%)	​
Immunosuppressant, n (%)	0	10 (18.2%)	12 (66.7%)	25 (65.8%)	
Rituximab, n (%)	0	2 (3.6%)	1 (5.6%)	2 (5.3%)	

eGFR, estimated glomerular filtration rate; urinary RBC count; urinary red blood cell count; M, mesangial hypercellularity; E, endocapillary hypercellularity; S, segmental glomerulosclerosis; T, tubular atrophy/interstitial fibrosis; C, cellular/fibrocellular crescents; *p < 0.05 compared with the RAS inhibitor group; ^#^P < 0.05 compared with glucocorticoid ± immunosuppressant group.

### 4.3 Primary outcomes

**FIGURE 2:**
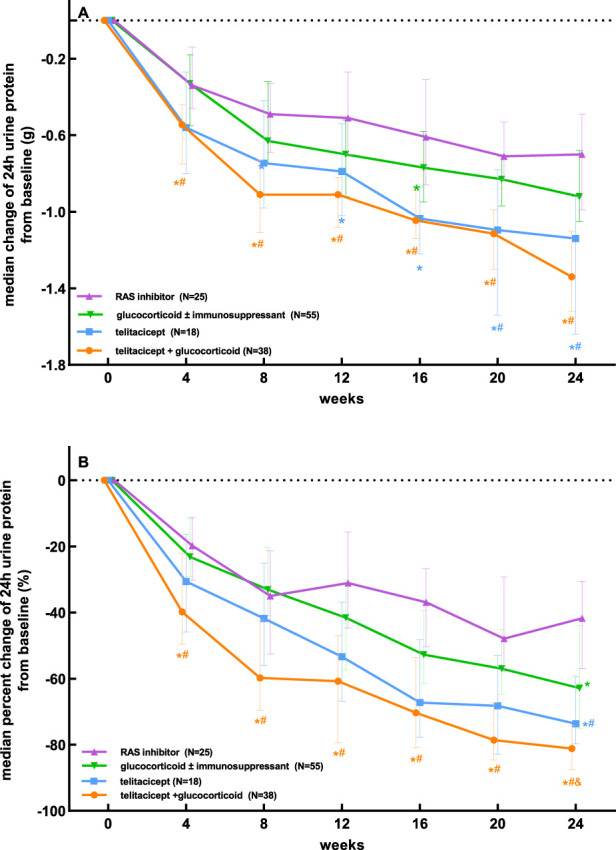
Change of proteinuria during follow-up period. **(A)** Median change of 24-h urinary protein from baseline (g). Data are expressed as median, upper quartile, and lower quartile. **(B)** Median percentage change of 24-h urinary protein from baseline (%). Data are expressed as median, upper quartile, and lower quartile. *p < 0.05 compared with RAS inhibitor group. #p < 0.05 compared with glucocorticoid ± immunosuppressant group. &p < 0.05 compared with telitacicept group.

Figure 2 and Table 2 show the madian change and median percentage change in UPE for each group. Starting at week 12, the telitacicept-based groups (both monotherapy and combination with glucocorticoids) showed consistently greater median reductions in UPE than the RAS inhibitor group (all P < 0.05; Table 2). At week 12, the median UPE reductions were 0.79 g (53.41%) in the telitacicept group and 0.90 g (60.76%) in the telitacicept + glucocorticoid group, compared to 0.51 g (31.06%) in the RAS inhibitor group. By week 24, the median reductions from baseline were 0.92 g (62.85%) in the glucocorticoid ± immunosuppressant group, 1.14 g (73.68%) in the telitacicept group, and 1.34 g (81.18%) in the telitacicept + glucocorticoid group, all of which were significantly greater than the median reduction observed in the RAS inhibitor group [0.70 g (41.75%); all P < 0.05).

**TABLE 2:** Median change (g) and median percentage change (%) in 24-h urinary protein compared with the baseline.

Follow-up time	RAS inhibitor (N = 25)	glucocorticoid ± immunosuppressant (N = 55)	telitacicept (N = 18)	telitacicept + glucocorticoid (N = 38)
Mean change in 24-h urinary protein from baseline (g)				
4-week	−0.34 (0.06,0.60)	−0.33 (0.07,0.70)	−0.56 (0.26,0.80)*	−0.55 (0.31,0.90)*^#^
8-week	−0.49 (0.24,0.80)	−0.63 (0.09,0.90)	−0.75 (0.40,0.99)	−0.91 (0.67,1.20)*^#^
12-week	−0.51 (0.19,0.71)	−0.70 (0.15,0.98)	−0.79 (0.54,1.07)*	−0.90 (0.73,1.32)*^#^
16-week	−0.61 (0.28,1.15)	−0.77 (0.48,1.15)	−1.03 (0.84,1.25)*	−1.04 (0.81,1.51)*^#^
20-week	−0.71 (0.45,0.90)	−0.83 (0.49,1.23)	−1.09 (0.78,1.58)*^#^	−1.11 (0.96,1.57)*^#^
24-week	−0.70 (0.40,1.07)	−0.92 (0.58,1.35) *	−1.14 (0.90,1.71)*^#^	−1.34 (1.00,1.73)*^#^
Mean percentage change in 24-h urinary protein from baseline (%)				
4-week	19.77 (4.45, 34.84)	23.16 (4.46, 47.62)	30.62 (15.29, 47.08)	39.83 (15.91, 59.02)*^#^
8-week	35.01 (20.12, 54.09)	33.05 (6.86, 51.49)	41.77 (24.32, 56.58)	59.77 (37.32, 71.76)*^#^
12-week	31.06 (9.59, 49.07)	41.56 (9.81, 66.32)	53.41 (36.73,68.39)*	60.76 (45.07, 83.03)*^#^
16-week	36.93 (20.39, 53.30)	52.76 (30.93, 75.49)	67.25 (46.08, 79.74)*	70.34 (49.10, 84.86)*^#^
20-week	47.89 (26.07, 62.10)	56.97 (33.37, 77.27)	68.23 (51.60, 82.90)*	78.61 (63.45, 85.33)*^#^
24-week	41.75 (28.60, 59.48)	62.85 (36.36,79.79)*	73.68 (59.20,85.26)*^#^	81.18 (67.82,90.01)*^#&^

Data are expressed as median value (upper quartile, lower quartile). *p < 0.05 compared with RAS inhibitor group; #p < 0.05 compared with glucocorticoid ± immunosuppressant group; & p < 0.05 compared with telitacicept group.

Furthermore, both the median change and percentage change in UPE were significantly greater in the telitacicept + glucocorticoid and telitacicept groups than in the glucocorticoid ± immunosuppressant group (all p < 0.05, Table 2; Figure 2). Additionally, at week 24, the median percentage change in UPE was significantly greater in the telitacicept + glucocorticoid group than in the telitacicept group [81.18% (67.82, 90.01) vs. 73.68% (59.20, 85.26), p = 0.026)].

### 4.4 Secondary outcomes

#### 4.4.1 Renal function

**FIGURE 3:**
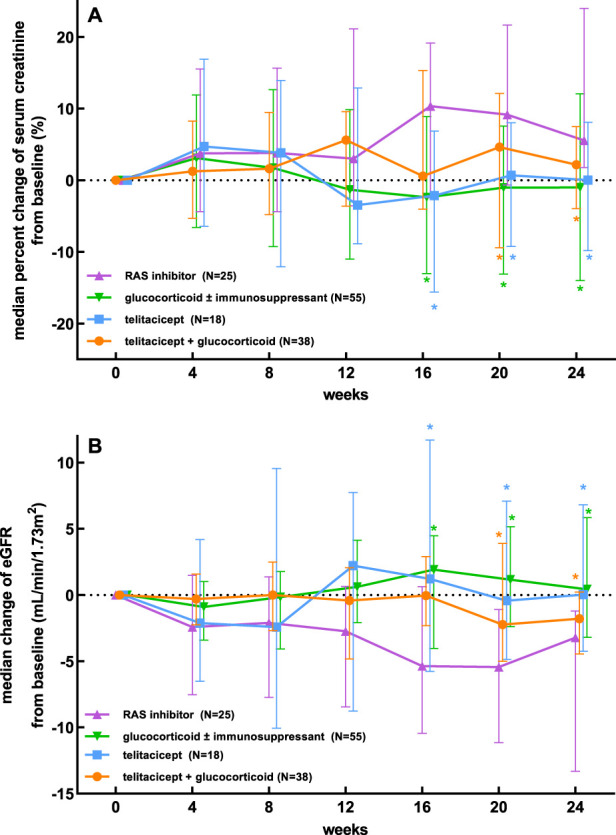
Change of renal function during follow-up period. **(A)** Median percentage change of serum creatinine from baseline (%) and median change of eGFR (ml/min/1.73 m^2^) from baseline **(B)**. Data are expressed as median, upper quartile, and lower quartile. *p < 0.05 compared with the RAS inhibitor group.

The median percentage change in Scr levels is shown in Figure 3A. In the telitacicept + glucocorticoid group, Scr at week 24 was 118.00 μmol/L (69.25, 161.25), which was not significantly different from baseline Scr [124.00 μmol/L (62.00, 156.25), P = 0.267]. The telitacicept group showed a slight, non-significant decrease in Scr at week 24 [84.00 μmol/L (65.25, 149.00)] compared to baseline [88.50 μmol/L (65.00, 143.50), P = 0.877]. Similarly, the glucocorticoid ± immunosuppressant group showed no significant change after 24 weeks treatment [93.00 μmol/L (67.00, 120.00) vs. 97.00 μmol/L (67.00, 142.00), P = 0.529]. In contrast, the RAS inhibitor group exhibited a significant increase in Scr at week 24 compared to baseline [93.00 μmol/L (73.50, 138.50) vs. 79.00 μmol/L (67.50, 132.00), P = 0.003]. Intergroup comparison revealed that at week 24, the median percentage change in Scr from the baseline was +5.57% (1.77, 23.43) in the RAS inhibitor group, which was significantly different from the glucocorticoid ± immunosuppressant group [-1.01% (−14.00, 12.07), P = 0.015], the telitacicept group [+0.02% (−9.81, 8.090, P = 0.036], and the telitacicept + glucocorticoid group [+2.17% (−3.97, 7.51), P = 0.040].

The RAS inhibitor group showed a progressive decline in eGFR, whereas the other three treatment groups exhibited minimal change in eGFR levels. At week 24, the median change in eGFR was −1.80 mL/min/1.73 m^2^ (−5.38, 2.20) from the baseline in the telitacicept + glucocorticoid group, −0.20 mL/min/1.73 m^2^ (−4.97, 8.04) in the telitacicept group, 0.42 mL/min/1.73 m^2^ (−7.00, 8.92) in the glucocorticoid ± immunosuppressant group, and −3.22 mL/min/1.73 m^2^ (−16.77, −0.70) in the RAS inhibitor group (Figure 3B). Compared to the RAS inhibitor group, the other three groups demonstrated significantly slower eGFR decline and greater stability (all P < 0.05).

#### 4.4.2 Urinary RBC count

After week 24, urinary RBC count decreased from 59.00 cells/μl (28.38, 170.00) to 7.15 cells/μl (3.05, 26.53) in telitacicept + glucocorticoid group (p = 0.022), from 167.95 cells/μl (93.75,341.45) to 27.10 cells/μl (9.38, 50.18) in telitacicept group (p = 0.020) and from 119.80 cells/μl (37.40,119.80) to 33.20 cells/μl (9.30, 66.80) in glucocorticoid ± immunosuppressant group (p = 0.041), while it showed no significant decrease in RAS inhibitor group [(from 71.90 cells/μl (9.80,228.65) to 42.50 cells/µl (14.05, 219.10), p = 0.764)].

**FIGURE 4:**
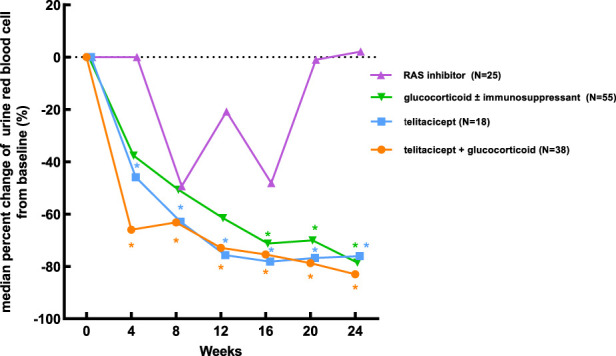
Change of hematuria during follow-up period. Median percentage change of urinary red blood cell count from the baseline. Data are expressed as median values without error bars. Error bars were omitted for skewed data to avoid visual distortion caused by excessively wide interquartile ranges and to ensure optimal graphical readability. *p < 0.05 compared with RAS inhibitor group. #p < 0.05 compared with glucocorticoid ± immunosuppressant group.

Intergroup comparison revealed that, starting at week 4, the median percentage reduction in urine RBC count was significantly greater in the telitacicept + glucocorticoid and telitacicept groups compared to the RAS inhibitor group (all P < 0.05, Figure 4). Furthermore, the glucocorticoid ± immunosuppressant group demonstrated a significantly greater reduction in urine RBC count compared to the RAS inhibitor group at weeks 20 and 24 (p = 0.002, p < 0.001).

### 4.5 Subgroup analysis

#### 4.5.1 Subgroup analysis by baseline UPE

**FIGURE 5:**
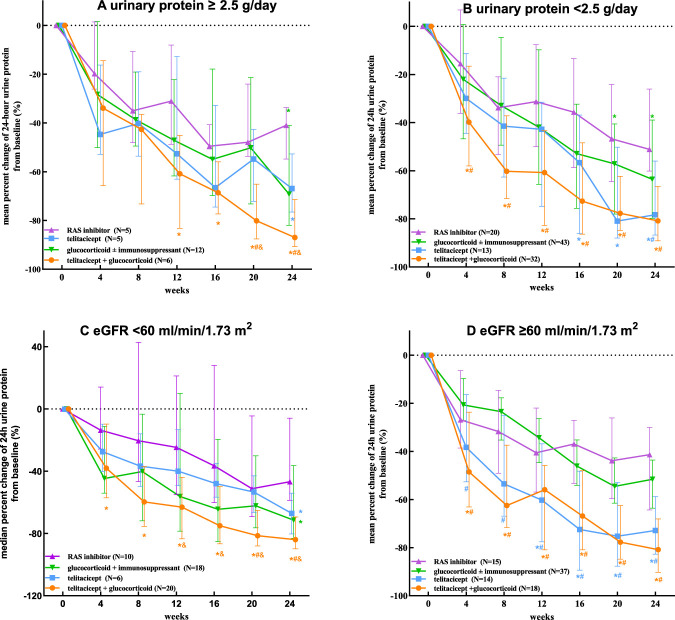
Subgroup analysis of median percentage changes in 24-h urinary protein from baseline (%). **(A)** In the subgroup with baseline urinary protein ≥2.5 g/d. **(B)** In the subgroup with baseline urinary protein <2.5 g/d. **(C)** In the subgroup with baseline eGFR <60 ml/min/1.73 m^2^. **(D)** In the subgroup with baseline eGFR ≥60 ml/min/1.73 m^2^. Data are expressed as median, upper quartile, and lower quartile. *p < 0.05 compared with RAS inhibitor group. #p < 0.05 compared with glucocorticoid ± immunosuppressant group. &p < 0 0.05 compared with telitacicept group.

In the subgroup with baseline UPE ≥2.5 g, the telitacicept + glucocorticoid group exhibited significantly greater median reductions in UPE at 24 weeks than in RAS inhibitor group, glucocorticoid ± immunosuppressant group and telitacicept group [86.99% (71.44, 90.74) vs. 40.97% (33.65, 54.81), p = 0.004; vs. 69.19% (41.05, 81.98), p = 0.042; vs. 67.40% (52.72, 78.87), p = 0.043]. The median reduction in UPE in the glucocorticoid ± immunosuppressant group and telitacicept group were higher than that in RAS inhibitor group (p = 0.047, 0.034, respectively; Figure 5A). No statistically significant difference was observed between the glucocorticoid ± immunosuppressant and telitacicept groups.

In the subgroup with baseline UPE <2.5 g, the median reduction in UPE from baseline at 24 weeks was 80.86% (66.54, 89.11) in the telitacicept + glucocorticoid group, 74.49% (63.21, 81.20) in the telitacicept group, and 58.49% (38.30, 79.69) in the glucocorticoid ± immunosuppressant group compared with a median change of 51.12% (26.06, 59.48) in the RAS inhibitor group (p < 0.001, <0.001, = 0.029). Compared to the glucocorticoid ± immunosuppressant group, telitacicept (with or without glucocorticoid) groups also showed a larger median percentage reduction in UPE (all p < 0.05). However, the efficacies of telitacicept + glucocorticoid group and telitacicept group were comparable (Figure 5B).

#### 4.5.2 Subgroup analysis by baseline eGFR

In the subgroup with baseline eGFR <60 ml/min/1.73 m^2^, the median percentage change in UPE was greater in the telitacicept + glucocorticoid group than in the telitacicept group and the RAS inhibitor group at week 8 [59.77% (36.40, 78.41) vs. 35.33% (10.74, 43.80), p = 0.035; vs. 25.14% (−27.83, 52.76), p = 0.010], week 12 [62.67% (43.20, 83.48) vs. 36.84% (12.77, 44.70), p = 0.015; vs. 28.38% (−3.30, 48.14), p = 0.002], week 16 [72.52% (49.62, 86.37) vs. 39.74% (32.77, 56.26), p = 0.029; vs. 40.39% (−18.46, 60.84), p = 0.003], week 20 [79.92% (64.50, 86.28) vs. 47.55% (41.25, 67.87), p = 0.010; vs. 51.22% (30.61, 71.54), p = 0.002], and week 24 [82.69% (67.32, 89.80) vs. 59.35% (52.72, 76.81), p = 0.029; vs. 46.77% (20.46, 57.62), p < 0.001]. Meanwhile, the glucocorticoid ± immunosuppressant group also achieved substantial UPE reduction at week 20 and week 24, with median percentage decreases of 63.06% (32.70, 80.87) and 76.35% (38.36, 83.48) from baseline, respectively. Nevertheless, the proteinuria reduction in this group remained significantly lower than that observed in the telitacicept + glucocorticoid group at the corresponding time points (p = 0.031 and p = 0.039, respectively; Figure 5C).

In the subgroup with baseline eGFR ≥60 ml/min/1.73 m^2^, the median decrease in UPE was significantly greater in the telitacicept (with or without glucocorticoid) groups than in other groups. At week 24, the median percentage reduction in UPE from baseline was significantly greater in both telitacicept + glucocorticoid group and telitacicept group compared to the glucocorticoid ± immunosuppressant group and RAS inhibitor group [80.86% (67.82, 90.33) and 75.78% (67.23, 82.33) vs. 51.61% (38.83, 76.51) and 41.33% (29.47, 62.19), all p < 0.05]. However, no statistically significant difference in efficacy was found between the two telitacicept-based regimens. Compared with the RAS inhibitor group, although the median percentage decline in UPE in the glucocorticoid ± immunosuppressant group showed a better trend, there was no statistical difference between the two groups [51.61% (38.83, 76.51) vs. 41.33% (29.47, 62.19), p = 0.120; Figure 5D].

### 4.6 Treatment efficacy

The clinical efficacy evaluation was performed for all patients, as shown in Table 3. Primary efficacy endpoints were complete remission (CR), partial remission (PR), and overall remission (OR). We defined CR as UPE ≤0.3 g and eGFR decline ≤10% from baseline. PR required a ≥50% reduction in UPE from baseline and eGFR decline ≤10%, without meeting the CR criteria. The OR were 68.42% (26/38, including 11 CR and 15 PR) in the telitacicept + glucocorticoid group, 72.22% (13/18, including 5 CR and 8 PR) in the telitacicept group, 43.64% (24/55, including 8 CR and 16 PR) in the glucocorticoid ± immunosuppressant group, and 36.00% (9/25, including 1 CR and 8 PR) in the RAS inhibitor group. The OR rates in both the telitacicept + glucocorticoid group and the telitacicept group were significantly higher than those in the RAS inhibitor and glucocorticoid ± immunosuppressant groups, suggesting that telitacicept may offer a clinical advantage in inducing urinary protein remission.

**TABLE 3:** Clinical remission rate during follow-up period.

N (%)	telitacicept + glucocorticoid (N = 38)	telitacicept (N = 18)	RAS inhibitor (N = 25)	glucocorticoid ± immunosuppressant (N = 55)
CR	11 (28.95%)	5 (27.78%)	1 (4.00%)	8 (14.55%)
PR	15 (39.47%)	8 (44.44%)	8 (32.00%)	16 (29.09%)
OR	26 (68.42%)	13 (72.22%)	9 (36.00%)	24 (43.64%)

CR, PR, and OR, by treatment group at 24 weeks; CR, complete remission; PR, partial remission; OR, overall remission.

### 4.7 Safety

Throughout the follow-up period, all adverse events (AEs) were either mild or moderate in severity, and no deaths or serious adverse events occurred in any of the four groups (Table 4). The incidence of AEs was similar between the telitacicept group (33.3%) and the RAS inhibitor group (32.0%). The most common AEs in the telitacicept group were urinary tract infections and injection site reactions. The RAS inhibitor group primarily exhibited urinary tract infection. The incidence of AEs was comparable between the glucocorticoid ± immunosuppressant (69.7%) and telitacicept + glucocorticoid (60.5%) groups, and both were higher than that in the RAS inhibitor group. These two groups had similar profiles of common AEs, primarily urinary tract infections, abnormal leukocyte counts, and respiratory tract infections. Among all the enrolled patients, a total of 26 (19.11%) urinary tract infections were reported, all of which were mild. Eighteen cases were asymptomatic, while 8 cases presented with mild lower urinary irritation symptoms. All cases were resolved with oral antibiotics without treatment interruption. Notably, injection site reactions were specific to the telitacicept-based groups. A total of 11 such reactions were reported, all presenting as mild local erythema and tenderness. These reactions resolved within 3–5 days with local management, requiring no adjustment to the treatment regimen. Abnormal liver function and new-onset diabetes were observed only in glucocorticoid ± immunosuppressant group.

**TABLE 4:** Adverse events.

Events, n (%)	RAS inhibitor (N = 25)	glucocorticoid ± immunosuppressant (N = 55)	telitacicept (N = 18)	Telitacicept + glucocorticoid (N = 38)	P
Total adverse events	9 (36.0)^#^	36 (69.7)	6 (33.3)^#^	23 (60.5) ^&^	0.005
Severe AE	0	0	0	0	​
Respiratory infection	2 (8.0)	3 (5.5)	0	2 (5.3)	0.704
Urinary tract infection	6 (24.0)	15 (27.3)	2 (11.1)	3 (7.9)^#^	0.086
Gastrointestinal discomfort	0	3 (5.5)	0	1 (2.6)	0.469
Injection site reactions	0	0	4 (22.2)*^,#^	7 (18.4)*^,#^	0.006
Liver injury	0	2 (3.6)	0	0	0.393
Abnormal leukocyte count	0	7 (12.7)	0	4 (10.5)	0.128
Constipation	0	2 (3.6)	0	1 (2.6)	0.677
Tremor	0	1 (1.8)	0	1 (2.6)	0.791
Acne	0	0	0	1 (2.6)	0.458
Insomnia	0	2 (3.6)	0	1 (2.6)	0.677
Allergic reaction	0	0	0	0	​
Joint pain	0	1 (1.8)	0	1 (2.6)	0.791
Osteoporosis	0	1 (1.8)	0	1 (2.6)	0.791
Hypotension	2 (8.0)	0	0	0	0.029
Impaired glucose tolerance	1 (4.0)	0	0	0	0.215
Hyperkalemia	2 (8.0)	0	0	0	0.029
New-onset diabetes	0	1 (1.8)	0	0	0.686

*p < 0.05 compared with RAS inhibitor group. ^#^p < 0.05 compared with glucocorticoid ± immunosuppressant group. & p < 0.05 compared with telitacicept group.

## 5 Discussion

In this retrospective cohort of high-risk IgAN patients, telitacicept group resulted in a median proteinuria reduction of 73.68%, while combination therapy with low-dose glucocorticoids further enhanced this improvement, with a median reduction of 81.18%. Notably, this combination therapy appears particularly effective in IgAN patients presenting with more severe proteinuria and impaired renal function. These findings suggest that telitacicept, either alone or in combination with glucocorticoids, may represent a promising therapeutic option for the management of IgAN in the future.

For IgAN patients with UPE ≥1 g and high risk of disease progression, there is still a lack of standardized treatment protocols. Protecting renal function in such patients and controlling disease progression are urgent clinical challenges. The 2025 KDIGO guidelines clearly state that, for patients with IgAN at risk of renal function progression, management should address both non-specific drivers of nephron loss and disease-specific drivers, underscoring the importance of precise targeting and comprehensive management (Rovin et al., 2025). Glucocorticoids can suppress localized inflammatory responses in the renal tissues. Previous studies have shown that a 6-month full-dose glucocorticoid treatment reduces proteinuria and renal failure events, while increasing the risk of SAEs such as mortality and severe infections. Low-dose glucocorticoids have similar efficacy but do not cause SAEs, suggesting that they may be a treatment option for IgAN patients (Lv et al., 2022). In recent years, many studies have confirmed that IgAN is closely associated with the “four-hit” hypothesis, mucosal immune dysregulation, and abnormal B-cell class switching and homing. Collectively, these mechanisms drive immune complex deposition, local renal inflammation, renal tissue damage, and functional decline.

Telitacicept, a recombinant TACI-Fc fusion protein, exerts its therapeutic effect by simultaneously blocking BLyS and APRIL, thereby inhibiting B-cell activation and differentiation, reducing the production of Gd-IgA1, and suppressing immune complex formation (Dhillon, 2021; Xie et al., 2022; Cai et al., 2023). The phase II clinical trial results of telitacicept demonstrated that at 24 weeks, urinary protein in the 240 mg and 160 mg treatment groups decreased by 49% and 25% from baseline (Lv et al., 2023). Emerging evidence supports the efficacy of telitacicept in IgAN. A study comparing telitacicept plus low-dose glucocorticoids versus MMF plus low-dose glucocorticoids in IgAN patients showed that the telitacicept group achieved a 62.5% reduction in proteinuria and demonstrated superior safety and renal protective effects (He et al., 2025). A retrospective study involving 19 centers in China demonstrated that telitacicept safely and significantly reduces proteinuria while stabilizing renal function. Subgroup analysis within this study indicated no significant difference in proteinuria reduction between telitacicept group and telitacicept combined with glucocorticoids or immunosuppressors group (Liu et al., 2024). However, another chinese multicenter retrospective study involving IgAN patients treated with 160 mg telitacicept monotherapy or telitacicept plus glucocorticoids found that both regimens significantly reduced proteinuria, and combination therapy with glucocorticoids or MMF resulted in more pronounced reductions and higher remission rates (Zeng et al., 2026). The comparative efficacy of telitacicept combined with immunosuppressants or glucocorticoids remains controversial, with discrepant conclusions reported across different studies. Given the urgent clinical requirements for proteinuria reduction and remission achievement, optimal dose selection and combination therapy strategies for telitacicept warrant further investigation.

The combination of telitacicept with glucocorticoids represents an exploratory therapeutic strategy based on pharmacological synergy. Glucocorticoids exert their effect by nonspecifically suppressing immune cell activation and proliferation, as well as reducing the release of pro-inflammatory factors, thereby controlling local glomerular inflammation. On the other hand, telitacicept exerts its therapeutic effect by dual suppression of BAFF and APRIL signaling pathways, thereby inhibiting B-cell activation and proliferation, reducing autoantibody production, and can target and continuously inhibit the production of Gd-IgA1 mediated by B cells. The combination regimen aims to simultaneously intervene in glomerular inflammation and B-cell mediated immunopathology, with the goal of achieving greater reduction in proteinuria, slowing the decline in eGFR, and potentially even modifying disease progression. Additionally, this strategy may allow for a reduction in cumulative glucocorticoid exposure, thereby minimizing long term treatment related adverse effects.

Unlike the experiments mentioned above, all patients in our cohort received a fixed dose of 240 mg/week of telitacicept and completed the full 24-week treatment course. In this study. we found that at 24 weeks, the median UPE decreased by 81.18% in the telitacicept + glucocorticoid group, 73.68% in the telitacicept group, and 62.85% in the glucocorticoid ± immunosuppressant group, compared with only 41.75% in the RAS inhibitor group. This result indicates that for IgAN with UPE ≥1 g, telitacicept-based regimens (with or without glucocorticoids) had a significant advantage in reducing urine protein levels. Its efficacy is superior to that of RAS inhibitors and immunosuppressive therapy. Unlike studies that solely compare the efficacy of telitacicept and RAS inhibitors in IgAN, this study suggests that compared with conventional glucocorticoid immunosuppressant therapy, telitacicept-based regimens (with or without glucocorticoids) may achieve superior and more rapid reductions in proteinuria. Among them, the effect of the telitacicept + glucocorticoid group appeared particularly pronounced. As early as the 8 weeks, the median rate of reduction in UPE reached 59.77%, and by the week 24, it had reached 81.18%. However, the median rate of reduction in UPE in the glucocorticoid ± immunosuppressant group was only 62.85%.

Further subgroup analysis revealed that, in patients with baseline UPE ≥2.5 g or eGFR <60 ml/min/1.73 m^2^, the telitacicept + glucocorticoid group achieved a more pronounced median reduction in UPE compared with other treatment groups. These findings indicate that telitacicept combined with low-dose glucocorticoids may serve as an optimal therapeutic strategy for high-risk IgAN patients with progressive potential. Further analysis of baseline medication history confirmed that more than 60% of patients in the telitacicept and telitacicept + glucocorticoid groups had a prior history of glucocorticoid or conventional immunosuppressant administration. Despite these prior interventions, all patients still exhibited persistent proteinuria >1 g/day at baseline, consistent with clinical features of refractory IgAN. Importantly, these patients achieved significant and sustained proteinuria remission after receiving telitacicept-based therapy. Collectively, these results further demonstrate that telitacicept may offer unique clinical advantages for the treatment of high-risk and refractory IgAN patients who are unresponsive to conventional immunosuppressive therapy.

During the treatment period, compared with the continuously decreasing trend in eGFR observed in the RAS inhibitor group, the telitacicept + glucocorticoid group, telitacicept group, and glucocorticoid ± immunosuppressant group all maintained relatively stable renal function. We further observed significant differences among treatment regimens for alleviating hematuria. Specifically, the RAS inhibitor group showed no significant reduction in hematuria in progressive IgAN patients. In contrast, telitacicept and glucocorticoid ± immunosuppressant regimens effectively improved hematuria. At week 24, the improvement in hematuria was significant in the telitacicept-based regimens, with the urinary RBC count decreasing by 76.09% (65.53, 90.77) in the telitacicept group, by 83.00% (49.63, 96.84) in telitacicept + glucocorticoid group.

No SAEs or death occurred during this study. The most common AEs in the telitacicept group were injection-site reactions and urinary tract infections, with incidences similar to those in the RAS inhibitor group. This finding is consistent with the results of a phase II clinical trial of telitacicept for IgAN, further corroborating its stable safety profile (Lv et al., 2023). However, the incidence of AEs was higher in the telitacicept + glucocorticoid and glucocorticoid ± immunosuppressant groups. Apart from the unique injection site reactions of telitacicept, urinary and respiratory tract infections were the main adverse reactions in both groups. Although all AEs in this study were mild or moderate, we still need to monitor for potential synergistic immunosuppressive effects when glucocorticoids are combined with biologics.

This study had several limitations. First, the retrospective cohort design, lack of randomization, limited sample sizes in treatment groups, and the higher baseline albumin level in the RAS inhibitor group may collectively affect statistical power, introduce selection bias, and influence the comparative efficacy outcomes. And our definition of high-risk IgAN was based primarily on proteinuria and eGFR, without incorporating the full multifactorial metrics of the International IgAN Prediction Tool, which may lead to less precise risk stratification. Second, the observation period of this study was only 24 weeks, which is insufficient to evaluate the long-term renal outcomes and sustained efficacy of telitacicept in IgAN patients. Third, this study lacked key pathogenic serum biomarkers (such as Gd-IgA1) and serial monitoring of immunoglobulin levels, which prevented an assessment of telitacicept’s efficacy at the disease mechanism level and a comprehensive evaluation of its potential immunological impact, including the risk of hypogammaglobulinemia. Finally, all enrolled patients in this study were of Asian ethnicity. It is well-established that the epidemiology, genetics, and clinical course of IgAN differ across racial and ethnic groups. Consequently, our findings may be specific to the Asian population, and caution is advised when extrapolating these results to other ethnicities. Future multi-ethnic studies are needed to validate the broader applicability of telitacicept globally. In summary, the long-term efficacy and safety of telitacicept alone or in combination with glucocorticoids in IgAN patients requires further validation in larger, higher-quality prospective randomized controlled trials for its broad clinical application.

## 6 Conclusion

In conclusion, this real-world retrospective study suggests that telitacicept may represent a promising treatment option for patients with IgAN. The findings indicate that compared to RAS inhibitor therapy or a glucocorticoid-immunosuppressive regimen, telitacicept-based treatment is associated with a greater reduction in proteinuria, alongside a milder decline in eGFR relative to the RAS inhibitor group. Notably, in patients with more severe proteinuria or impaired renal function, the combination of telitacicept with low-dose glucocorticoids provides superior proteinuria reduction compared with other regimens. These findings may provide further clinical evidence supporting the use of telitacicept in the management of IgAN. Nevertheless, given the exploratory and observational nature of the present retrospective study, these results warrant further validation in prospective randomized controlled trials.

## Data Availability

The data analyzed in this study is subject to the following licenses/restrictions: The datasets presented in this article are not readily available because The dataset is available upon reasonable request for academic or research purposes. Interested researchers may contact the corresponding author via email to request access. The dataset is subject to the following restrictions: Usage Restriction: The data may only be used for non-commercial, academic, or scientific research purposes. Confidentiality: Users must agree not to attempt to re-identify any individuals from the de-identified data. Redistribution Prohibition: The dataset may not be shared, distributed, or published in its raw form without prior written permission from the authors. Attribution Requirement: Any publications or presentations resulting from the use of this dataset must appropriately cite the original source publication. If these conditions are met, the raw data can be provided via email upon request. Requests to access the datasets should be directed to xugsh882003@163.com. Requests to access these datasets should be directed to Guoshuang Xu, xugsh882003@163.com.
